# Improvement of Fish Growth and Metabolism by Oligosaccharide Prebiotic Supplement

**DOI:** 10.1155/2022/5715649

**Published:** 2022-10-28

**Authors:** Wei Xu, Charles Greg Lutz, Christopher M. Taylor, Miriam Contin Ortega

**Affiliations:** ^1^Department of Life Sciences, College of Science and Engineering, Texas A&M University Corpus Christi, Corpus Christi, Texas, USA; ^2^Agricultural Center, Louisiana State University, Baton Rouge, Louisiana, USA; ^3^Department of Microbiology, Immunology & Parasitology, Louisiana State University Health Sciences Center, New Orleans, LA, USA

## Abstract

Finfish aquaculture is expected to continue to benefit from significantly improved fish diets, which are the source of energy to support the growth and health of fish. Strategies to enhance the transformation rate of dietary energy and protein to fish growth are greatly desired by fish culturists. Prebiotic compounds can be used as supplements to human, animal, and fish diets to populate beneficial bacteria in the gut. The goal of the present study is to identify low-cost prebiotic compounds with high efficacy in increasing the absorption of food nutrients by fish. Several oligosaccharides were evaluated as prebiotics in Nile tilapia (*Oreochromis niloticus*), one of the most widely cultured species in the world. Several parameters of the fish on different diets were evaluated, including feed conversion ratios (FCRs), enzymatic activities, expression of growth-related genes, and the gut microbiome. Two age groups of fish (30 days old and 90 days old) were used in this study. The results indicated that the addition of xylooligosaccharide (XOS), galactooligosaccharide (GOS), or XOS and GOS combination to the basic fish diet significantly decreased the feed conversion ratio (FCR) of the fish in both age groups. Both XOS and GOS decreased the FCR of 30-day-old fish by 34.4% compared to the fish on the control diet. In the 90-day-old fish group, XOS and GOS decreased the FCR by 11.9%, while the combination of the two prebiotics led to a 20.2% decrease in FCR compared to the control group. The application of XOS and GOS also elevated the production of glutathione-related enzymes and the enzymatic activity of glutathione peroxidase (GPX), indicating the enhancement of antioxidation processes in fish. These improvements were associated with significant changes in the fish gut microbiota. The abundance of *Clostridium ruminantium*, *Brevinema andersonii*, *Shewanella amazonensis*, *Reyranella massiliensis*, and *Chitinilyticum aquatile* were upregulated by XOS and GOS supplements. The findings of the present study suggested that the prebiotics would be more effective when they were applied to the younger fish, and the application of multiple oligosaccharide prebiotic compounds could result in a greater growth enhancement. The identified bacteria can be potentially used as probiotic supplements in the future to improve fish growth and feeding efficiency and ultimately reduce the cost of tilapia aquaculture.

## 1. Introduction

Advances in nutrition and feeding play essential roles in the sustained development of finfish aquaculture. How efficiently an aquaculture species can convert nutrients in the feed to body mass is a critical consideration in many perspectives. Maximizing feed conversion ratios (FCRs) reduces the amount of feed required in culture systems and ultimately minimizes the environmental impacts resulting from the unconsumed nutrients released from the system. Many internal and external factors can affect FCR in fish culture, such as feed ingredients, feeding methods, fish strains, fish physiology, and environment [1]. Improving FCR in aquaculture continues to be a priority; however, practical investigations in this area are difficult considering the complexity of internal and external factors.

Improvement of FCR on an individual basis can be achieved by increasing the net utilization of dietary inputs or limiting physical and metabolic activities [2]. Another factor that influences FCR for larger numbers of fish involves the timing and severity of mortality during a production cycle [2, 3].Given that limiting physical and metabolic activities often negatively affects fish health during culture, it is generally more feasible to focus on enhancing the utilization of fish feed. Such efforts are ongoing in many aquaculture fish species. The most common approach is to optimize dietary ingredients so that the digestibility and utilization of key nutrients can be maximized [4, 5]. However, the improvement of FCR through such formulation adjustment is typically marginal. Therefore, the improvement of FCR through enhancing nutrients' gastrointestinal (GI) absorption is increasingly important in aquaculture.

It is known that bacteria colonize internal and external surfaces of all metazoans including fish [6]. A number of metabolic processes in fish, including GI functions, have been shown to be associated with their microbial communities [7–9]. Changing GI microbiomes in individuals can dramatically change their physiological performance. Research previously conducted on human obesity demonstrated that transplantation of GI microbiomes from an obese human patient to the GI of a germ-free mouse resulted in increased body mass and signs of obesity in the mouse [10]. Studies on the influence of GI microbiomes on productivity in livestock and poultry have been widely performed since the development of the next-generation sequencing (NGS) technique. The GI microbiomes in these farmed animals are not only dynamically associated with their diets but also can be used as indicators reflecting the physiological conditions of the animals [11–14].

Compared to studies in mammals, fish microbiome research continues to lag well behind [6]. Most of the studies in fish were performed in zebrafish (*Danio rerio*) as a biomedical model [15, 16]. Current studies on fish GI microbiomes focus on aquaculture species, such as Siberian sturgeon (*Acipenser baerii*) [17], grouper (*E. coioides*) [18], rainbow trout (*O. mykiss*) [19], and Atlantic salmon (*S. salar*) [20]. The majority of these fish studies focused on the GI microbiomes with certain types of external or internal stresses. However, mechanistic studies in how GI microbiome changes influence the physiological status of fish are very rare. Therefore, methods to utilize the GI microbiota as tools to improve the quality of aquaculture fish species remain elusive.

Nonetheless, approaches to the manipulation of fish GI microbiomes toward beneficial communities have drawn increasing research interest. Several popular strategies have been used in some aquaculture fish species. Applications of prebiotics for fish health and growth performance have demonstrated great efficiency [21]. Prebiotics has been defined as non-digestible food ingredients that can regulate the growth of certain bacteria in GI tracts and consequently improve host health and growth [22]. The effect of prebiotic application has also been confirmed in Siberian sturgeon, in which the application of arabinoxylooligosaccharide prebiotics successfully stimulated the growth of beneficial bacteria, Lactobacillaceae, in the GI tract [23]. Many more prebiotics have been used in fish production, including insulin, fructooligosaccharides, short-chain fructooligosaccharides, mannanoligosaccharides, transgalactooligosaccharides, galactooligosaccharides, xylooligosaccharides, and isomaltooligosaccharides [24]. These components can be used to control the balances of a variety of bacterial families, which can be beneficial to fish growth and health.

Nile tilapia (*Oreochromis niloticus*) was used in this study based on its importance as an aquaculture species in the United States and globally. Originally from Africa and the Middle East, tilapias are cultured worldwide and have become the second most farmed fish behind carps. The annual worldwide production of farmed tilapia exceeds 4.8 million tonnes with an estimated market value of over 8.2 billion US dollars [25]. Tilapia production has been leading freshwater aquaculture in many tropical countries and areas, such as China, the Philippines, Indonesia and Thailand, which are major suppliers [25]. The production of tilapia in the United States consistently increased before year 2003; however, dramatically declined since that time. Compared to 2003 when the production of tilapia in the U.S. hit the highest level in history (>320,000 tonnes), this value was reduced by 45% (<180,000 tonnes) in 2013. Low profit caused by the cost of diet in tilapia farming is the main reason causing the reduction of tilapia culture in the U.S.

To improve the utilization rate of fish diet, several oligosaccharides were supplemented to a commercial fish diet as prebiotic components. The impacts of the prebiotic components on the FCR of fish and the dynamics of gut microbiomes were evaluated. Results from this study will help advance an understanding of the importance of the fish gut microbiota in fish growth. These findings may contribute to the development of supplementary products for fish feed to advance the economic and environmental sustainability of finfish aquaculture production.

## 2. Materials and Methods

### 2.1. Diet Preparation

The prebiotic compounds were mixed with the fish diet AquaXcel Starter 5014 (0.8 mm) purchased from Cargill Animal Nutrition (Wayzata, MN). Each of the four tested prebiotic compounds, fructooligosaccharide (FOS), isomaltooligosaccharide (IOS), xylooligosaccharide (XOS), and galactooligosaccharide (GOS), was mixed separately with the diet at 5% (5 g of probiotics in every 100 g diet). Crisco Pure Vegetable Oil (Parsippany, NJ) at 1% (v/w) was used to maintain the attachment of prebiotic powder to the surface of the diet. Accordingly, an additional diet was prepared utilizing vegetable oil without prebiotics as well as a control diet with no oil added, for a total of six diets. All diets were kept at 4 °C at all times except for the time of fish feeding.

Based on initial results, a second trial was conducted utilizing 5% XOS, 5% GOS, and, separately, a combination of XOS and GOS with each at 2.5% by weight, as well as the control diet with vegetable oil.

### 2.2. Animal Housing and Experimental Design

Fingerlings (*O. niloticus*) were purchased from the Louisiana Specialty Aquafarm (Tangipahoa, LA) and maintained at the Louisiana State University Agricultural Center (LSU AgCenter), Aquaculture Research Station (Baton Rouge, LA). Permit approval for possession of Nile tilapia was obtained from the Louisiana Department of Wildlife and Fisheries. The fish housing and handling protocol were reviewed and approved by the LSU Agricultural Center Institutional Animal Care & Use Committee (IACUC).

In the first trial, each of the diets described above was fed to one-month-old *O. niloticus* fingerlings (2 g average weight) for a period of 90 days. Five replicate 45 L tanks were utilized for each diet, for a total of 30 tanks in a single 1850 L recirculating system with a reservoir sump and a biomechanical floating bead filter. Each tank had independent water supply. Initially, fish were fed to apparent satiation three times daily during the first 14 days, and twice daily thereafter, following the grower's recommendation. Any noticeable unconsumed feed was removed after approximately one hour after each feeding. The total weight of feed fed was recorded daily for each tank, as was any mortality. The temperature was maintained at 27 ± 2°C with a light/dark cycle 12 h:12 h. The water quality was monitored weekly throughout the study to comply with recommended values for Nile tilapia culture (pH 7.5-8, 220 + 20 mg/l alkalinity, 280 + 15 mg/l total hardness, and 0.3-0.4 g/l chlorides).

Based on results from the first trial, the second trial was conducted utilizing the four diet treatments described above and 3-month old juvenile fish (90 g average weight) stocked in 280 L tanks. Two 2750 L recirculating systems were used for the growth trial, each with eight tanks, a reservoir sump, and a biomechanical bead filter, and each system included two replicate tanks per treatment. Similar to Trial I, the tanks in Trial II had independent water supplies. The growth trial lasted for 84 days, and as in the first trial, fish were fed to apparent satiation twice daily, and any noticeable unconsumed feed was removed after approximately one hour. The total weight of feed fed was recorded daily for each tank, as was any mortality. The temperature was maintained at 28 ± 2°C, and water quality was monitored and adjusted weekly. The fish larvae from each trial were randomly selected from the fish farm. The first and second trials on fish larvae were independent of each other, and no comparisons were made between the results from the two trials.

### 2.3. Growth Evaluation

At the end of each growth trial, the fish were anesthetized with 50 mg/L tricaine methane-sulfonate (MS-222) buffered with NaHCO_3_ solution (pH 7.2-7.4). The length (cm) and weight (g) of each fish were measured followed by blood withdrawal. The body mass index (BMI) of each fish is calculated following the equation, BMI = weight (kg)/[height (m)]^2^. Feed conversion ratio (FCR) is calculated for each tank of fish with the following equation: FCR = total weight of applied feed (g)/gain of fish weight (g). The weight of the diet fed to the fish in each tank was recorded daily, and the total weight of the diet during the experiment was calculated. The total wet weight of the fish in each tank was measured before and after the experiment to calculate weight gain for each replicate tank within a dietary treatment. The total bodyweight of the fish in each tank was measured before and after the experiment to calculate the gain of body weight for the fish in each tank.

### 2.4. Blood and Organ Collections

Upon the completion of the fish measurement, the anesthetized fish was used for blood collection via the caudal vein with 21 Ga syringes [26]. The tubes used for blood collection contained 25 USP unit heparin (50 *μ*L 500 units/mL heparin solution), and the syringes for blood withdrawal were rinsed with 500 units/mL heparin solution briefly. Approximately 1 mL of blood was collected from each fish individual using this method. Thereafter, the fish was dissected and approximate 200 mg of liver was collected and preserved in Trizol reagent (Thermo-Fisher) for RNA extraction. The whole content of GI tract was collected and preserved in the lysis buffer from the DNeasy PowerSoil Pro Kit (Qiagen) followed by the bacterial DNA extraction as outlined in the protocol of the kit.

### 2.5. Gut Microbiome Analysis

The purified gut microbial DNA samples were sent to the Microbial Genomics Resource Group (MGRG) within the LSU Health Sciences Center School of Medicine in New Orleans. The DNAs were first amplified by a pair of commonly used bacterial 16S rRNA gene PCR primers (Supplementary Data 1) targeting the highly variable V4 region across bacterial species [27, 28]. The amplified products were subjected to the Illumina MiSeq high-throughput sequencer for sequencing. Data analyses with the sequences were also performed by the MGRG. Briefly, all the sequences obtained from the Illumina sequencer were preprocessed to remove reads with low-quality, ambiguous bases, and short lengths (<240 bp). The reads passing the quality control were processed through the DADA2 algorithm [29] implemented in QIIME 2 [30].Taxonomic assignment was performed using the SILVA database v138 [31]. Identified bacteria with known names of species, genera, and families were used to construct heatmaps to demonstrate their relative abundance.

### 2.6. Quantification of Growth-Related Transcripts

The transcripts of selected growth-related genes were quantified using quantitative PCR (qPCR) analysis with liver tissues. The RNA isolation was performed using TRIzol followed by DNA removal using TURBO DNA-free™ Kit and RNA cleaning with Qiagen RNeasy Mini Kit. The cDNA of each RNA sample was synthesized using the Invitrogen SuperScript IV Reverse Transcriptase system (Thermo-Fisher). The transcript levels of the genes encoding glutathione S-transferase (*gst*), glutathione peroxidase (*gpx*), glutathione-disulfide reductase (*gsr*), growth hormone receptor II (*ghr2*), catalase (*cat*), superoxide dismutase (*sod*), fatty acid synthase (*fas*), acetyl-CoA carboxylase *β* (*acacb*), and carnitine palmitoyltransferase 1 (*cpt1*) were analyzed using qPCR on a QuantStudio 3 Real-Time Thermocycler (Applied Biosystems, Waltham, MA) with the following cycling conditions: 50 °C for 2 min; 94 °C for 2 min; and 40 temperature cycles including 30 s of 94 °C, 30 s of 55 °C, and 30 s of 72 °C. The primers for qPCR are listed in Supplementary Data 1. The Ct values of all selected genes in all samples were normalized using the Ct values from a housekeeping gene, glyceraldehyde-3-phosphate dehydrogenase (*gapdh*), and relative transcript levels of each gene in livers from the fish with different diets were calculated using the 2^-*ΔΔ*Ct^ method [32].

### 2.7. Activities of Growth and Immune-Related Enzymes

The enzymatic analyses were performed on four enzymes, including pyruvate kinase, glutathione peroxidase, superoxide dismutase, and catalase, using the EnzyChrom™ enzyme activity kits (BioAssay Systems, Hayward, CA). Blood withdrawn from each fish (1 mL) was centrifuged to separate the serum from the blood cells. 500 mL serum from each blood sample was collected and used for the enzymatic analysis following the manufacturers' instructions for the kits. The activity of each enzyme with treatment was calculated using the standard curves.

### 2.8. Statistical Analyses

Basic descriptive statistics for survival- and growth-related traits in each trial were calculated in Microsoft Excel. Prior to the statistical analyses, all data generated from different qPCR, enzymatic, and microbiome assays were all tested using the Shapiro–Wilk test [33] for the normality tests and Bartlett's test [34] for the homogeneity tests. All data generated from this study were confirmed to be normally distributed with good homogeneities and eligible for the ANOVA tests. Multiple comparisons between treatment and control groups were performed using one-way ANOVA and Tukey's tests with least-squares means. One-way ANOVA tests were performed with the data of fish growth (weight, length, BMI, and FCR), qPCR, and enzymatic analyses. All analyses were done in R.

## 3. Results

### 3.1. *O. niloticus* growth Performance with Prebiotic Supplements

**Figure 1:**
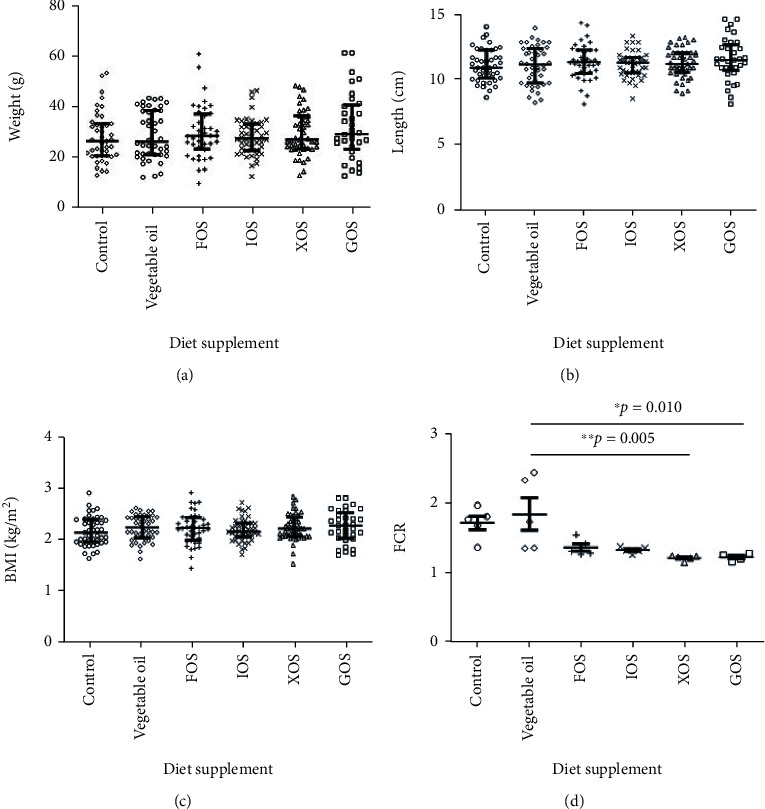
Growth performance of the one-month larval *O. niloticus*. The fish growth performance was evaluated by measuring the wet weight (a) and body length (b) and calculating the BMI of the fish (c). The FCR was also calculated (d). ^∗^0.01 < *p* < 0.05; ^∗∗^*p* < 0.01; *n* = 33 − 45 for (a)–(c) and 5 for (d).

**Figure 2:**
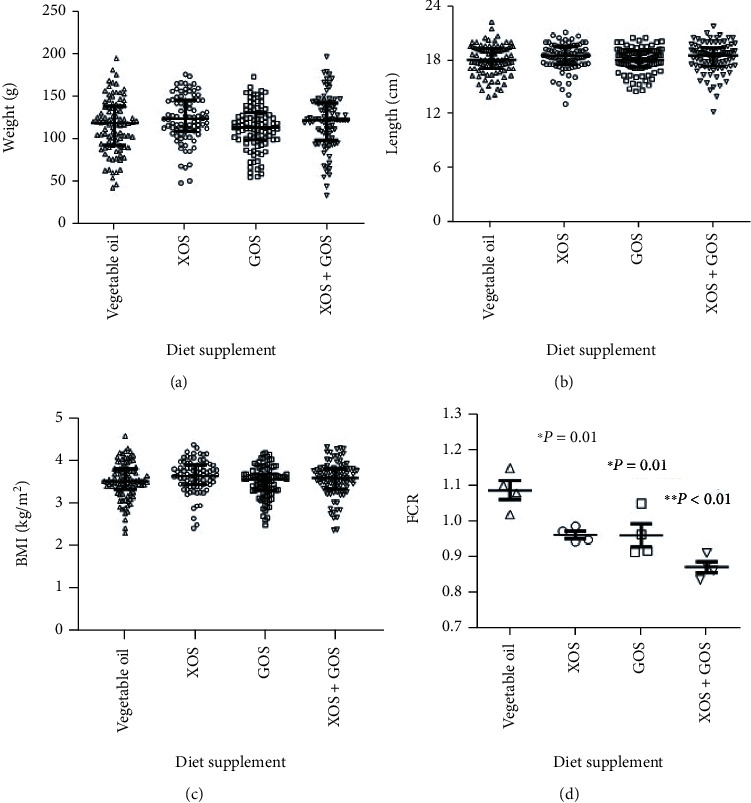
Growth performance of the three-month juvenile *O. niloticus*. Parameters, including wet weight (a), body length (b), and the BMI (c) of the fish, were used to assess the growth performance. FCR was calculated to estimate the nutrient transformation from fish diets to fish growth (d). ^∗^0.01 < *p* < 0.05; ^∗∗^*p* < 0.01; *n* = 33 − 45. *n* = 86 − 95 for (a)–(c) and 4 for (d).

Four prebiotic compounds were tested with one-month-old *O. niloticus* larvae in the first growth experiment. Vegetable oil was used to mix the prebiotics with the basic fish diet. In the first trial, survival did not differ significantly among treatments (*p* = 0.81), indicating that FCR and growth values were valid for statistical comparisons and not simply artifacts of density effects resulting from differential survival. There was no significant difference in weight (Figure 1(a)), body length (Figure 1(b)), BMI (Figure 1(c)), or FCR (Figure 1(d)), between the fish larvae fed with the basic diet only and those fed on a basic diet containing vegetable oil. Therefore, the basic diet with vegetable oil was used as the control diet for the fish in the other experiment (Figure 2). Like the vegetable oil, adding FOS, IOS, XOS, or GOS to the fish diet did not significantly improve the fish weight, body, length, or BMI (Figures 1(a)–1(c)). However, the XOS and GOS reduced the FCR from 1.83 ± 0.24 (basic diet + vegetable oil) to 1.20 ± 0.02 (*p* = 0.005) and 1.20 ± 0.03 (*p* = 0.01), respectively (Figure 1(d)). The total weight of the diet fed in each tank during the trial showed less diet was used for the fish supplemented by prebiotics. Compared to the control diet, the average weight of FOS, IOS, XOS, and GOS diet for each tank reduced by 12.3, 12.8, 19.3, and 19.0%, respectively (Supplementary data 2). No significant difference was found between the control and vegetable oil diets.

The combination of XOS and GOS was tested in the second fish growth experiment (Figure 2). The juvenile *O. niloticus* used in this study were three months old. Similar to the results from one-month-old fish, the three-month juveniles did not gain more bodyweight (Figure 2(a)), length (Figure 2(b)), or BMI (Figure 2(c)) when fed with XOS, GOS, or the combination of the two prebiotics compared to those fed on the control diet. However, XOS, GOS, and their combination reduced the FCR of the basic diet from 1.09 ± 0.03 to 0.96 ± 0.01 (*p* = 0.01), 0.96 ± 0.03 (*p* = 0.01), and 0.87 ± 0.02 (*p* = 0.0001), respectively (Figure 2(d)). Similarly, the application of XOS, GOS, and XOS + GOS to the diet significantly reduced the amount of diet fed per tank during the trial compared to the vegetable oil diet with a decrease of 17.8, 17.3, and 27.9%, respectively (Supplementary data 2). Direct effects and interactions resulting from the two separate recirculating systems were not statistically significant and were not included in subsequent data analysis. As in the first trial, survival did not differ significantly among treatments (*p* = 0.43), indicating FCR and growth values were valid for statistical comparisons.

### 3.2. Gene Transcript Analyses

**Figure 3:**
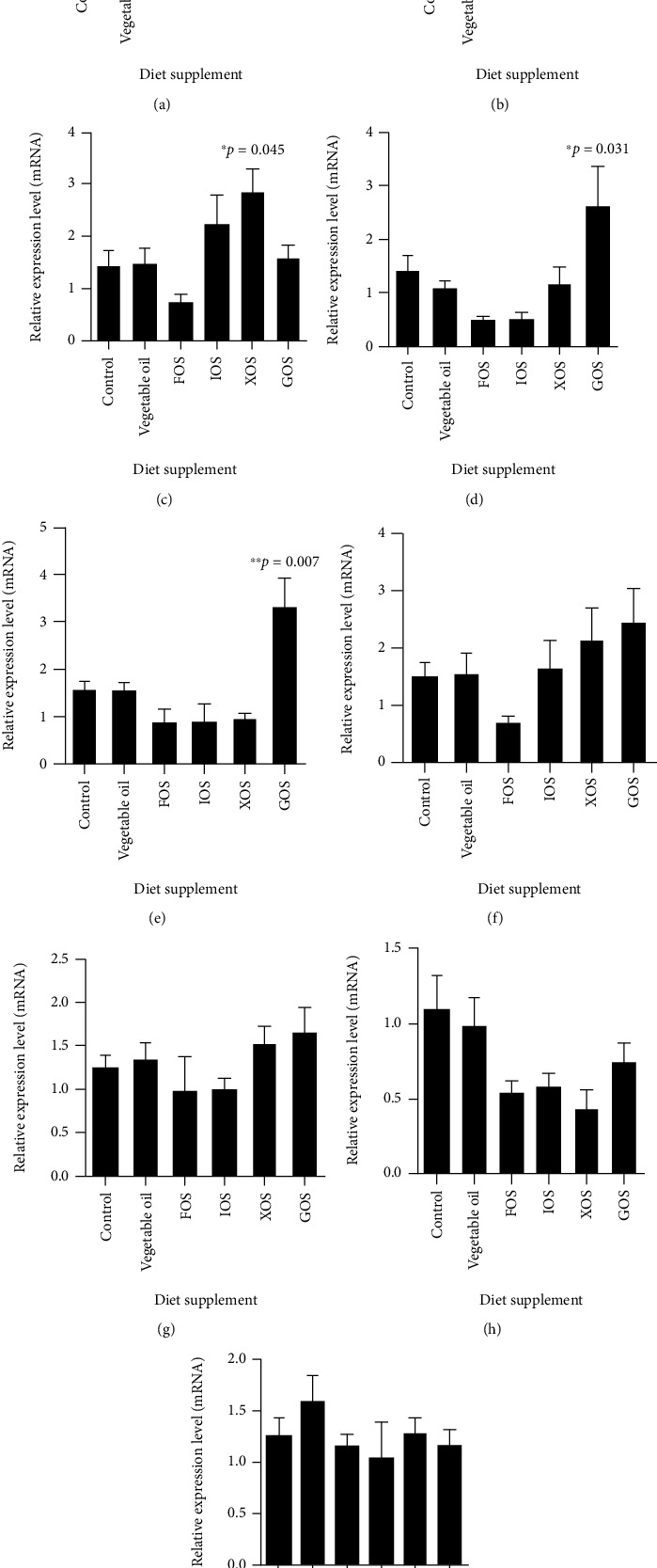
Transcript analysis of fish metabolism-related genes. The relative expression levels of glutathione S-transferase (a), glutathione peroxidase (b), glutathione-disulfide reductase (c), growth hormone receptor II (d), catalase (e), superoxide dismutase (f), fatty acid synthase (g), acetyl-CoA carboxylase *β* (h), and carnitine palmitoyl transferase 1 (i) were tested using qPCR. ^∗^0.01 < *p* < 0.05; ^∗∗^*p* < 0.01; *n* = 6 − 24.

Similar to the fish growth performance results, supplementing vegetable oil to the fish basic diet did not alter the expression level of any genes tested in this study. The expression levels of glutathione S-transferase encoding gene (*gst*) in livers of fish from the IOS, XOS, and GOS treatment were significantly higher than those with the control diet, with 3.50 ± 0.73 (*p* = 0.018), 3.34 ± 0.54 (*p* = 0.034), and 3.70 ± 0.77 (*p* = 0.047) fold changes, respectively (Figure 3(a)). The transcripts of another glutathione-related protein, glutathione peroxidase (*gpx*), also increased in the fish fed with XOS and GOS with fold changes 2.32 ± 0.48 (*p* = 0.006) and 2.08 ± 0.23 (*p* = 0.043), respectively (Figure 3(b)). In addition, the glutathione-disulfide reductase encoding gene (*gsr*) was only upregulated by XOS with a 2.91 ± 0.43 fold (*p* = 0.045) change compared to control (Figure 3(c)). Two other genes encoding growth hormone receptor II (*ghr-2*) and catalase (*cat*) were only upregulated by GOS. The transcript of *ghr-2* in GOS treated group was 2.63 ± 0.71 (*p* = 0.031) times of that in the control diet group (Figure 3(d)). The transcription level of *cat* in GOS group was 3.32 ± 0.60 (*p* = 0.007) folds of that in control group (Figures 3(e) and 3(f)). Other tested genes did not show notable up- or downregulation in any prebiotic treatment groups compared to the control diet (Figures 3(g)–3(i)).

### 3.3. Enzyme Activities in Fish Sera

**Figure 4:**
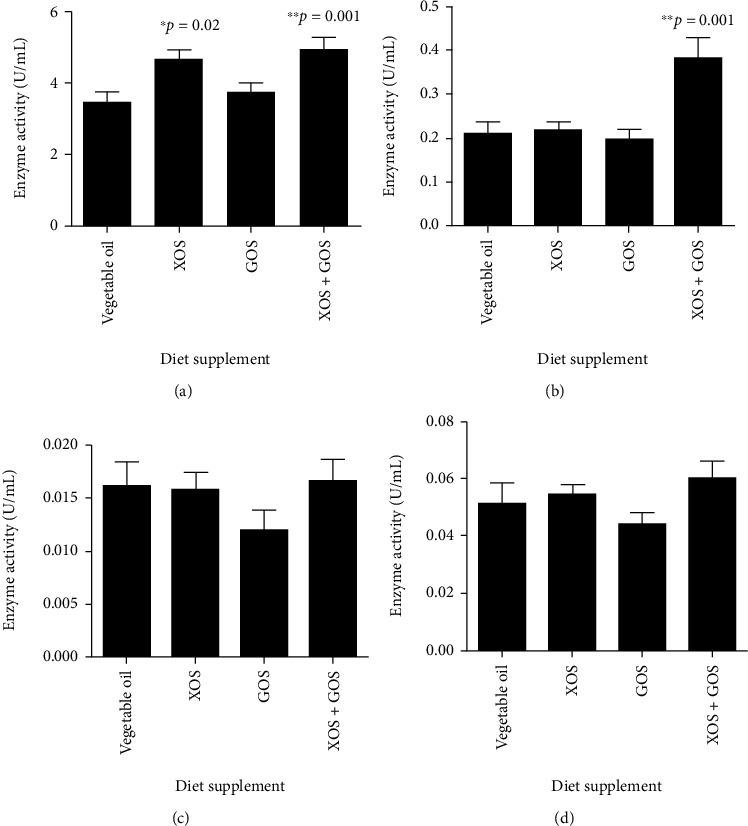
Enzymatic analysis. The activities of metabolism-related enzymes, including glutathione peroxidase (a), pyruvate kinase (b), superoxide dismutase (c), and catalase (d), were tested. ^∗^0.01 < *p* < 0.05; ^∗∗^*p* < 0.01; *n* = 10 − 12.

Activities of selected enzymes were tested with the serum samples from the three-month juvenile fish in the second trial. The activity of glutathione peroxidase (GPX) in the fish treated with the control diet was 3.49 ± 0.25 U/mL. The GPX activities in XOS and XOS + GOS diet groups were 4.69 ± 0.30 and 4.97 ± 0.44 U/mL, respectively (Figure 4(a)), which were significantly higher than the control group (*p* = 0.017 and 0.001, respectively). The GOS did not enhance the GPX activity in fish sera compared to the control diet (Figure 4(a)). The pyruvate kinase activity was only enhanced by the diet with addition of XOS + GOS from 0.21 ± 0.03 U/mL (control) to 0.38 ± 0.05 U/mL (*p* = 0.001) (Figure 4(b)). The two other enzymes, superoxide dismutase (Figure 4(c)) and catalase (Figure 4(d)), were not significantly affected by any diet.

### 3.4. Microbiome Change in Response to Prebiotics

**Figure 5:**
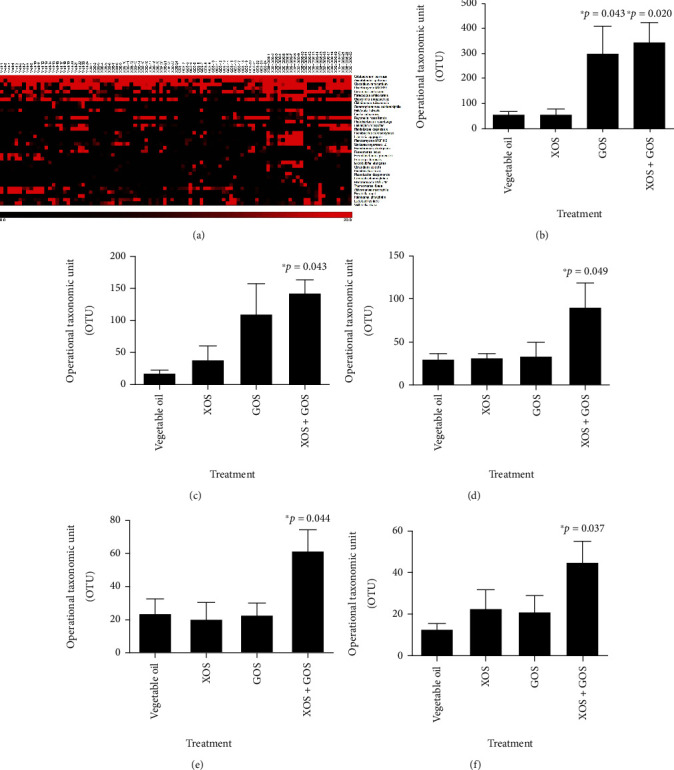
Bacteria species identified by 16S rDNA sequencing analysis. The identified bacteria with known species names were demonstrated by the heatmap with the color representing the species abundance (a). The bacteria species with upregulated abundance in XOS, GOS, or XOS + GOS include *Clostridium ruminantium* (b), *Brevinema andersonii* (c), *Shewanella amazonensis* (d), *Reyranella massiliensis* (e), and *Chitinilyticum aquatile* (f). ^∗^0.01 < *p* < 0.05; *n* = 24.

The bacterial species, genera, orders, families, or classes were identified based on the sequencing data. There were 35 bacterial species that were identified from this study (Figure 5(a)). Among these species, five were found to be upregulated by the supplement of XOS, GOS, or XOS + GOS. *Clostridium ruminantium* was significantly higher in the fish fed with GOS or XOS + GOS (Figure 5(b)). *Clostridium ruminantium* in control (vegetable oil) was 57 ± 11, while in GOS and XOS + GOS, it was elevated to 299 ± 108 (*p* = 0.043) and 348 ± 75 (*p* = 0.020), respectively. Four other bacteria species, including *Brevinema andersonii* (Figure 5(c)), *Shewanella amazonensis* (Figure 5(d)), *Reyranella massiliensis* (Figure 5(e)), and *Chitinilyticum aquatile* (Figure 5(f)), were found upregulated in XOS + GOS diet treatment compared to the control diet. *B. andersonii*, *S. amazonensis*, *R. massiliensis*, and *C. aquatile* changed from 18 ± 5, 30 ± 7, 24 ± 9, and 13 ± 3 with control diet to 142 ± 21 (*p* = 0.043), 91 ± 28 (*p* = 0.049), 62 ± 12 (*p* = 0.044), and 45 ± 11 (*p* = 0.037) with XOS + GOS diet, respectively. In addition, 159 genera, 58 orders, 94 families, and 42 classes were identified from this analysis. The top candidates of genera are shown in Supplementary Data 3.

## 4. Discussion

In recent decades, efforts in the development of prebiotics for aquaculture have been more focused on oligosaccharides, which are often present in plants. The efficacies of some oligosaccharide compounds have been confirmed in many aquacultured fish. Oligosaccharide supplemented diets were reported to cause diarrhea in rainbow trout and Atlantic salmon (Refstie et al.; [35]). The application of mannanoligosaccharides (MOS) significantly decreased the apparent digestibility coefficient (ADC) of lipids while increasing the ADCs of protein, organic matter, and carbohydrates in red drum [36]. Results suggest that the performance and application of prebiotics can be improved to minimize negative effects on fish. The application of arabinoxylooligosaccharide prebiotics successfully stimulated the growth of beneficial bacteria, Lactobacillaceae, in the GI tract of Siberian sturgeon [23]. Other oligosaccharides have also been studied in fish production, including FOS, GOS, transgalactooligosaccharides (tGOS), XOS, and IOS with varied results [24].

Four oligosaccharides were evaluated in the present study, FOS, IOS, XOS, and GOS. Results of both fish growth experiments with different age groups of *O. niloticus* did not indicate significant differences in weight, body length, or BMI between different diet treatments. However, the total feed fed in XOS, GOS, and the combination XOS and GOS treatments was considerably less than in the control diet, resulting in significantly lower FCRs. In one-month-old fingerlings, XOS and GOS decreased FCR by 35.0% and 34.4%, respectively. In three-month-old juveniles, both XOS and GOS reduced the FCR by 12.0%. The combination of XOS and GOS decreased FCR even more, by 20.0% compared to the control diet. The combination of XOS and GOS improved FCR values compared to each one separately, although the statistical analysis did not indicate a significant difference (*p* values of XOS + GOS vs. XOS and XOS + GOS vs. GOS were 0.065 and 0.070, respectively). The addition of prebiotic compounds to fish diets demonstrated greater benefits in the first trial with younger fish, suggesting prebiotic supplementation may be of greater value during the early development stages of fish.

The addition of XOS or GOS enhanced the expression levels of several glutathione-related genes including *gst*, *gpx*, and *gsr*, which encode glutathione S-transferase (GST), glutathione peroxidase (GPX), and glutathione-disulfide reductase (GSR), respectively. Since glutathione is an antioxidant compound in animals, protecting cellular components from reactive oxygen species (ROS), the upregulation of those glutathione-related proteins suggested the activation of the antioxidation pathway by these prebiotic compounds. In this pathway, GST promotes the conjugation of glutathione to toxicants. GPX catalyzes the oxidation of glutathione, which is associated with the removal of hydrogen peroxide (H_2_O_2_) from cells to reduce the levels of peroxide radicals [37].While GPX removes H_2_O_2_, the reduced glutathione (GSH) is oxidated to become glutathione disulfide (GSSG). As a reductase, GSR reduces GSSG to GSH with the hydrogen ion (H^+^) provided by the nicotinamide adenine dinucleotide phosphate (NADPH). GSH is known to play a key role in detoxification by forming the GS-conjugated construct, which is catalyzed by GST, the third glutathione-related enzyme in this study. The upregulation of the genes encoding these three proteins suggests that XOS and GOS are involved in antioxidation and detoxification in *O. niloticus*.

The activity of GPX in serum samples of fish from different prebiotic treatment groups was also measured. As a biomarker for antioxidation, GPX demonstrated enhanced activities in fish fed XOS (34% increase) and XOS + GOS (42% increase) compared to that in the fish from the control group. This confirmed the function of XOS in antioxidation in *O. niloticus*, as suggested by the qPCR result. Although GOS did not show a significant effect on the enzyme activity of GPX (Figure 4(a)), the supplement of GOS along with XOS appeared to further increase GPX activity in fish. Interestingly, the activity of pyruvate kinase was also improved by the combination of XOS and GOS supplements (Figure 4(b)). Pyruvate kinase is known to be an enzyme catalyzing the generation of pyruvate and adenosine triphosphate (ATP) from phosphoenolpyruvate (V. [38]) as the last step of glycolysis. Since ATP is the major energy source for cells, the function of pyruvate kinase is considered to be related to energy generation in organisms, which explains the decreased FCR in XOS + GOS treated fish.

The dynamics of fish GI microbiomes are known to be critical in the maintenance of overall fish health [39], metabolism [40], and physiological condition [28]. Among fish GI microbiome studies, many have focused on how diet influences bacterial community composition. Bacterial communities influence the utilization of various nutrients in the feed. More efficient utilization of nutrients will improve FCR values in cultured fish, which consequently enhances environmental and economic sustainability in aquaculture. In addition, the fish with different prebiotic supplement demonstrated some changes in eating behavior. The fish feed was added to each tank progressively with careful observation to avoid overfeeding, which could affect the accuracy of FCR calculation. Fish supplied with the prebiotic compounds consumed less food than the control groups. It is very likely to be the consequence of the shift of gut microbiota caused by the prebiotics. To this end, investigations focusing on the fish GI microbiome are increasingly recognized as critical steps toward the improvement of fish production in aquaculture.

In the present study, the numbers of many bacterial species were found to be enhanced in the gut of the fish with XOS + GOS treatment. The top five bacteria were *C. ruminantium*, *B. andersonii*, *S. amazonensis*, *R. massiliensis*, and *C. aquatile*. *Clostridium* species have been identified as a predominant cluster of gut bacteria in many species [41]. They are also in the guts of many fish species, working as symbionts [42]. A previous study on the prebiotic effects of arabinoxylan oligosaccharides (AXOS) in Siberian sturgeon (*Acipenser baerii*) showed an upregulation of *C. ruminantium* in the gut microbial population with AXOS supplementation in a fish diet [17]. *B. andersonii* was also previously reported as a major gut microbial species in *O. niloticus* when the fish was fed with probiotics [43]. Similarly, the Atlantic salmon (*Salmo salar*) fed with an alginate supplemented diet carried more *B. andersonii* in the gut (S. [44]). *B. andersonii* is a beneficial bacterial species to many organisms since it is necessary for the production of butyrate, which is a critical short-chain fatty acid for the health of the digestive system (S. [44, 45]).

The other three bacteria species, *S. amazonensis*, *R. massiliensis*, and *C. aquatile*, have not been widely studied in fish digestive tracts and metabolism. Limited studies suggest that *Shewanella* species may contribute to the synthesis of omega-3 fatty acids in freshwater fish [46] and the protection of fish from infection of the *Betanodavirus* [47] and *Vibrio* [48]. The symbiotic growth of *Reyranella* bacteria in the gut of tropical gar, *Atractosteus tropicus*, was believed to have positive effects on the survival of adult fish in an adverse environment [49]. The potential roles of *C. aquatile* in fish have not been understood despite the strong chitinolytic activity of *Chitinilyticum* bacteria. Many *Chitinilyticum* bacteria were isolated from freshwater shrimp ponds, including *C. aquatile* [50, 51].

The application of selected oligosaccharides in the fish diet improved FCR over the basic diet in *O. niloticus*. More efforts should be made in the modification of supplemented prebiotics in fish diets. First, to allow ingestion, prebiotic components must be maintained in the feed for a certain amount of time without being dissolved in water. Compared to the usage of prebiotics in humans and livestock, the delivery of prebiotic compounds in aquaculture is challenging due to the unique aquatic environment. Secondly, although the prebiotic compounds in fish diets need to remain integrated without being dissolved in the surrounding water, the compounds have to be soluble in water once the feed is taken by the fish. This is critical to the efficient absorption of prebiotics. Therefore, the solubility of prebiotic compounds in water must also be considered. Finally, prebiotic compounds should remain functional for a relatively long time to provide a consistent and sustainable effect on the host. Considering the complexities of fish GI environments, a prebiotic compound with more resistance to enzymatic digestion and consumption by microbes in the GI tract is desired to provide a more sustained effect on nutrient absorption.

## 5. Conclusions

The present study demonstrated the benefit of prebiotic compounds in the improvement of FCR in *O. niloticus* culture. Compared to fish in the control diet treatment, fingerling and juvenile *O. niloticus* demonstrated similar growth performance while consuming a reduced amount of feed supplemented with XOS and GOS prebiotic compounds. The addition of these compounds enhanced the production of glutathione-related proteins, which suggested they contributed to antioxidation and detoxification in *O. niloticus*. The upregulation of GPX activity in fish sera associated with XOS and GOS supplementation supported the role of these two prebiotics in host detoxification. These genetic and physiological changes appear to be attributed to changes in gut microbiota. The bacterial species with enhanced profiles associated with XOS and GOS can potentially be used as probiotic candidates in *O. niloticus* production in the future.

## Data Availability

The sequencing data used to support the findings of this study are available from the corresponding author upon request.

## Supplementary Materials

Supplementary Materials Supplementary Data 1. Primers for qPCR. Supplementary Data 2. Total weight of diet fed in each tank of fish. Supplementary Data 3. Identified genera of bacteria from 16S rDNA sequencing.
