# Supplementary material for: Improvement of Fish Growth and Metabolism by Oligosaccharide Prebiotic Supplement
Source: Aquac Nutr. 2022 Oct 28;2022:5715649. doi: 10.1155/2022/5715649 (PMC9973164; doi:10.1155/2022/5715649)
Supplement: Supplementary Materials — Supplementary Data 1. Primers for qPCR. Supplementary Data 2. Total weight of diet fed in each tank of fish. Supplementary Data 3. Identified genera of bacteria from 16S rDNA sequencing. [file 5715649.f1.docx]

**SUPPLEMENTARY DATA**

**Supplementary Data 1**. Primers for qPCR

| **Target gene** | **Organism** | **Direction** | **Sequence** | **Purpose** |
| --- | --- | --- | --- | --- |
| ***gapdh**** | *O. niloticus* | Forward | 5’- GTG TCA ACC ACG AGA AGT ATG A-3’ | qPCR |
|  |  | Reverse | 5’- CAG TAG AAG CAG GGA TGA TGT T-3’ | qPCR |
| ***gst*** | *O. niloticus* | Forward | 5’- CTC TGA CCA CTG AAC TCA AAC T-3’ | qPCR |
|  |  | Reverse | 5’- GGC TTT GAC ACT GGG TCT ATC-3’ | qPCR |
| ***gpx*** | *O. niloticus* | Forward | 5’- GGA ACC TTA CAA GCG CTA CA-3’ | qPCR |
|  |  | Reverse | 5’- CAA ACC CAG GCC TGC TAT AA-3’ | qPCR |
| ***gsr*** | *O. niloticus* | Forward | 5’- CAG AGG GAA GGA GAA TGT GAA G-3’ | qPCR |
|  |  | Reverse | 5’- CTT GAT GGC CAC AGC AAA TC-3’ | qPCR |
| ***ghr2*** | *O. niloticus* | Forward | 5’- GAA CCA CCA CCT GTC TTC TAA C-3’ | qPCR |
|  |  | Reverse | 5’- CTC AGA GGT TGC TGC ACA TAA-3’ | qPCR |
| ***cat*** | *O. niloticus* | Forward | 5’- GAA CTT GGC CGG GTT TCT A-3’ | qPCR |
|  |  | Reverse | 5’- CTT AGA TGA GGC GGT GAT GG-3’ | qPCR |
| ***sod*** | *O. niloticus* | Forward | 5’- CAA AGG GAG ACG TGA CAA CA-3’ | qPCR |
|  |  | Reverse | 5’- CAC CGT AAC AGC AGA CAT CTT-3’ | qPCR |
| ***fas*** | *O. niloticus* | Forward | 5’- CCT TGT GTG CCT TCA TCC A-3’ | qPCR |
|  |  | Reverse | 5’- CAG CAG CCT TTA GCT TGT AGT A-3’ | qPCR |
| ***acacb*** | *O. niloticus* | Forward | 5’- CTC AGT GGA TCA GGC CAA TTT A-3’ | qPCR |
|  |  | Reverse | 5’- AGG ACT CCG AGA AAG GAG TAT T-3’ | qPCR |
| ***cpt1*** | *O. niloticus* | Forward | 5’- GGT GTA GAA ACA GAC ACC CTT C-3’ | qPCR |
|  |  | Reverse | 5’- CTG CAG TAA GTG CTG CTA GTC-3’ | qPCR |
| **16S** | Bacteria | 515F | 5’-CCA TCT CAT CCC TGC GTG TCT CCG ACT CAG-3’ | Sequencing |
| **rDNA** |  | 909R | 5’- CCT ATC CCC TGT GTG CCT TGG CAG TCT CAG -3’ | Sequencing |

*The house-keeping gene used as an internal reference.


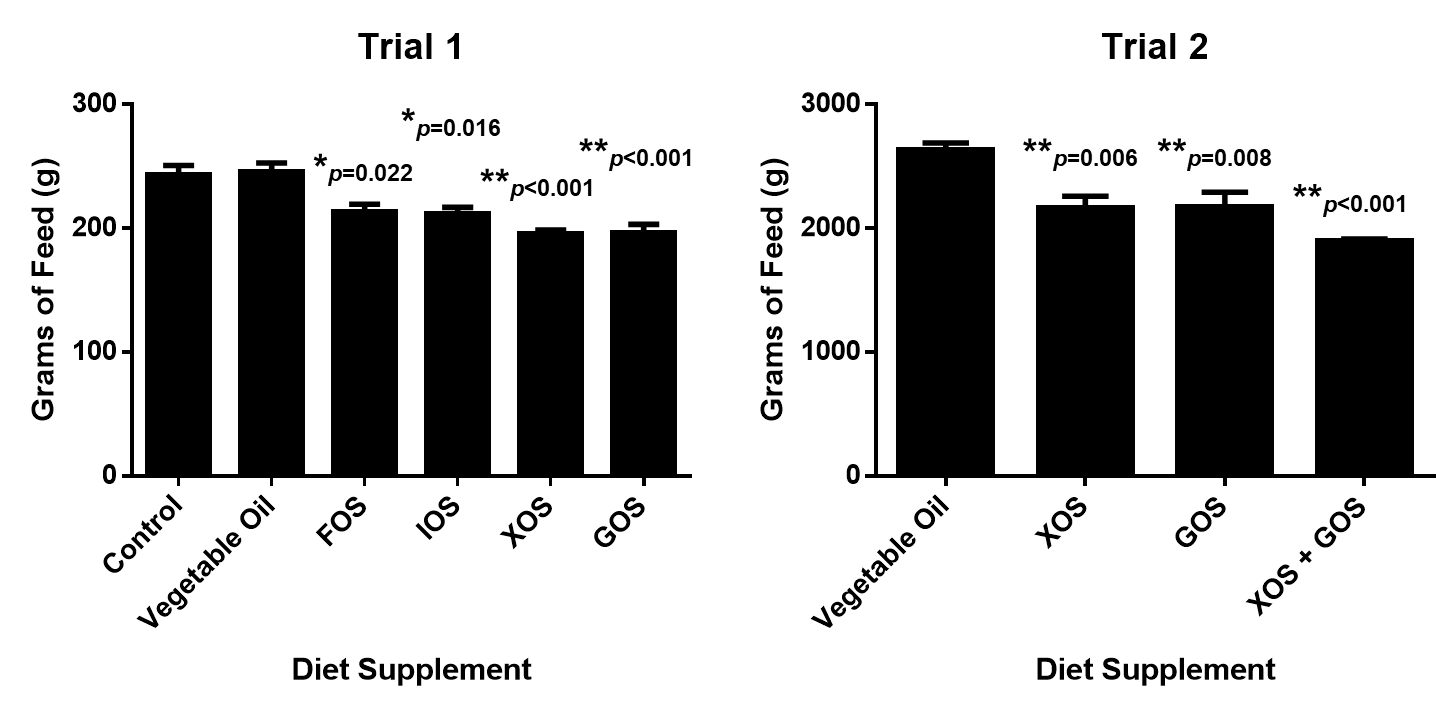


**Supplementary Data 2**. Total weight of diet fed in each tank of fish. The bars represent the average weights of the total diet fed in each tank during the experiment. In Trial 1, n=6. In Trial 2, n=4.


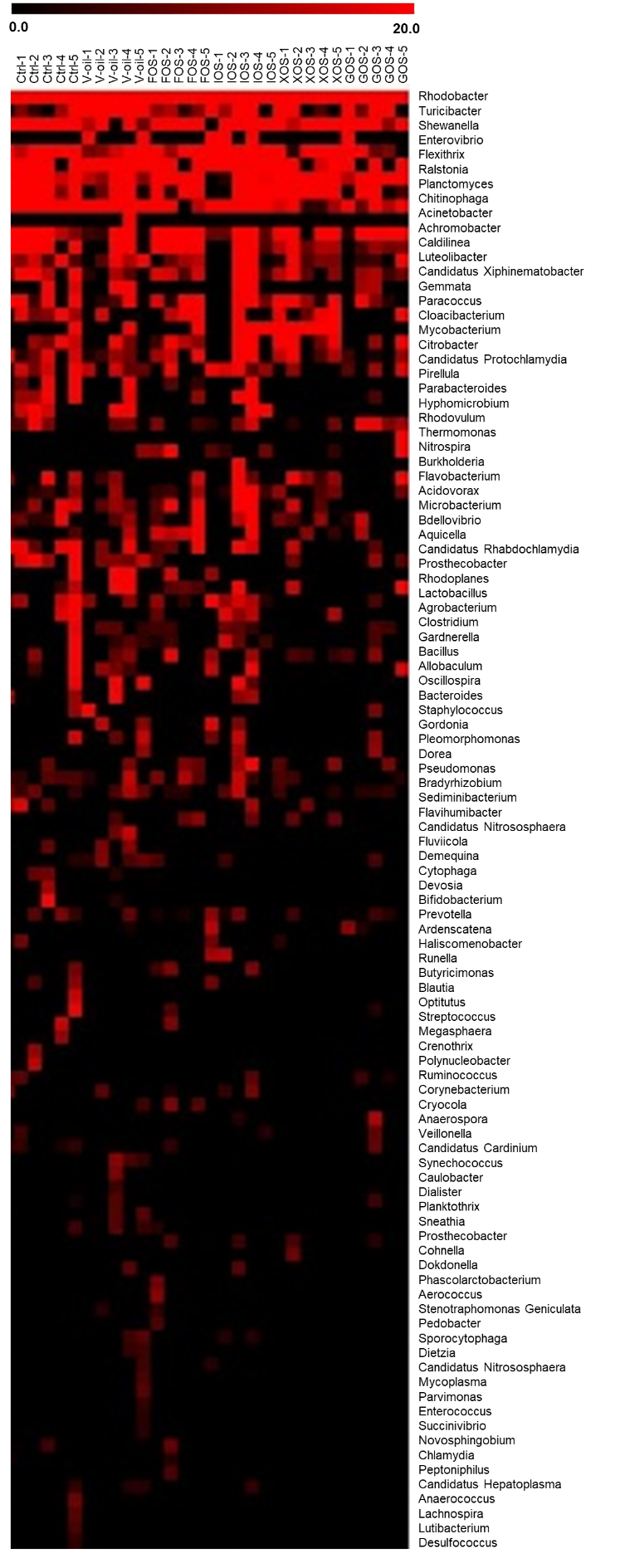


**Supplementary Data 3**. Identified genera of bacteria from 16S rDNA sequencing. Scale bar represents the range of OTUs.
